# Molecular Basis of Soybean Resistance to Soybean Aphids and Soybean Cyst Nematodes

**DOI:** 10.3390/plants8100374

**Published:** 2019-09-26

**Authors:** Surendra Neupane, Jordan M Purintun, Febina M Mathew, Adam J Varenhorst, Madhav P Nepal

**Affiliations:** 1 Department of Biology and Microbiology, South Dakota State University, Brookings, SD 57007, USA; surendra.neupane@sdstate.edu (S.N.); jordan.purintun@sdstate.edu (J.M.P.); 2 Department of Agronomy, Horticulture and Plant Science, South Dakota State University, Brookings, SD 57007, USA; febina.mathew@sdstate.edu (F.M.M.); adam.varenhorst@sdstate.edu (A.J.V.)

**Keywords:** α-SNAP, effectors, *GmPAD4*, *GmSHMT08*, induced susceptibility, *Rag* genes, *Rhg* genes, soybean pest resistance

## Abstract

Soybean aphid (SBA; *Aphis glycines* Matsumura) and soybean cyst nematode (SCN; *Heterodera glycines* Ichninohe) are major pests of the soybean (*Glycine max* [L.] Merr.). Substantial progress has been made in identifying the genetic basis of limiting these pests in both model and non-model plant systems. Classical linkage mapping and genome-wide association studies (GWAS) have identified major and minor quantitative trait loci (QTLs) in soybean. Studies on interactions of SBA and SCN effectors with host proteins have identified molecular cues in various signaling pathways, including those involved in plant disease resistance and phytohormone regulations. In this paper, we review the molecular basis of soybean resistance to SBA and SCN, and we provide a synthesis of recent studies of soybean QTLs/genes that could mitigate the effects of virulent SBA and SCN populations. We also review relevant studies of aphid–nematode interactions, particularly in the soybean–SBA–SCN system.

## 1. Introduction

**Figure 1:**
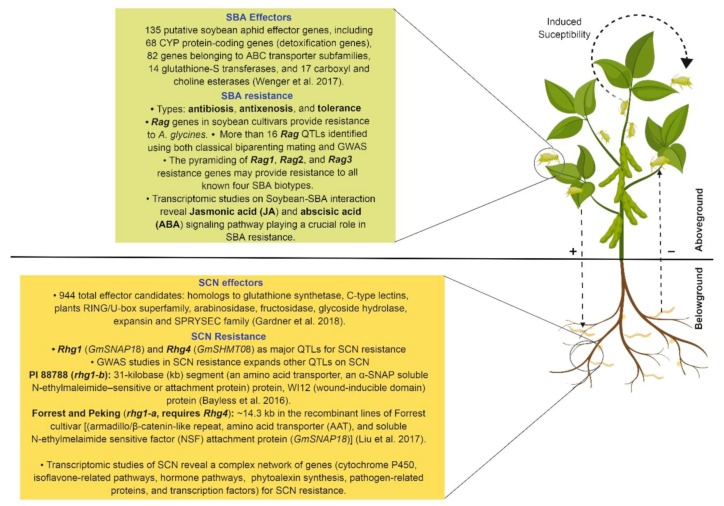
Overview of soybean resistance to soybean aphid (SBA) and soybean cyst nematode (SCN), as well as their interactions in soybean. Various SBA and SCN resistance components are shown in the projected green and yellow rectangular boxes, respectively. The circular arrow represents the process of induced susceptibility within conspecifics of soybean aphid. The dashed arrows represent the interactions between soybean aphid and SCN identified by the various studies discussed in this review. The (+) and (−) signs represent the positive and negative effects, respectively. This illustration was created using Biorender (https://app.biorender.com/). (CYP = cytochrome p450, QTL = quantitative trait loci, GWAS = genome-wide association studies, *Rag* = resistance to *Aphis glycines*, and α-SNAP = α soluble N-ethylmaleimide–sensitive factor attachment protein).

Soybean (*Glycine max* [L.] Merr.), a source of high-quality sugar, protein, and oil, is one of the most important crops worldwide [1]. The soybean aphid (SBA), *Aphis glycines* Matsumura (Hemiptera: Aphididae), and soybean cyst nematode (SCN), *Heterodera glycines* Ichinohe (Tylenchida: Heteroderidae), are common pests that cause significant losses in soybean production [2,3,4]. The soybean aphid is an aboveground pest that feeds on phloem sap, while the SCN infects soybean roots underground (Figure 1). Annual losses in US soybean production due to SBA and SCN are estimated to be approximately $4 billion and $1.3 billion, respectively [5,6,7]. The evolution of different SBA biotypes and SCN populations with virulent characteristics can decrease the efficacy of resistant cultivars [8,9]. Understanding SBA, SCN, and their interactions is necessary to develop and deploy durable host resistance in soybean. The major objective of this paper is to provide a thorough review on soybean resistance to SBA and SCN. Emphasis is placed on pest biology, the functions of effectors, molecular resistance mechanisms, and the interactions of SBA and SCN with one another.

## 2. Soybean Aphid

### 2.1. The Soybean Aphid Utilizes Soybean as a Secondary Host

The SBA is a heteroecious, holocyclic species that uses various species of buckthorn (genus *Rhamnus* L.) as a primary host and utilizes soybean as a secondary host [10]. It overwinters on buckthorn before emerging in the spring to produce several generations via sexual reproduction [11]. In the late spring or early summer, the aphids develop into alates (winged morphs) and migrate to soybean plants, where they feed primarily on the ventral surfaces of young leaves [12]. This results in plant stunting, leaf yellowing and wrinkling, reduced photosynthesis, poor pod fill, reduced seed size and quality, and yield reductions of up to 40% [13,14,15]. The aphids also act as a vector for various viruses (*Soybean mosaic virus*, *Alfalfa mosaic virus*, and *Bean yellow mosaic virus*) and facilitate sooty mold formations through the deposition of honeydew [5,16]. The SBA is native to East Asia and is considered to be a major pest in China, Japan, the Philippines, South Korea, Indonesia, Malaysia, Thailand, Vietnam, and Russia [17]. It has been present in North America since at least 2000, when it was first reported in the state of Wisconsin [2], and has also spread to Australia [18]. By 2009, the species had spread to the northeastern and Midwestern regions of the United States [19] and has since been found in thirty states and three Canadian provinces [20].

### 2.2. Soybean Aphids Have Developed Different Biotypes 

A biotype is an insect population that can survive and reproduce in cultivars developed for resistance to that same population [21]. It is a pseudo-taxonomic unit that classifies insect populations according to shared phenotypes and virulence to specific cultivars (reviewed in [22]). This term has been used for various insect species (e.g., *Mayetiola destructor* Say, *Schizaphis graminum* Rodani, *Nilaparvata lugens* Stål, and *Bemisia tabaci* Gennadius) [21]. Soybean aphids that are avirulent on any soybean plant that contains the *Rag* or other QTL loci are attributed to biotype 1 [23]. Biotype 1 is the predominant biotype in North America [24]. Biotype 2 (*Rag1* virulent) was discovered in Ohio in 2005, five years before the release of commercial *Rag1* cultivars [25]. Biotype 2 aphids were thought to be the predominant biotype in eastern North America [25], but various field tests have found that they are prevalent only in Ohio [9]. Biotype 3 aphids discovered in Indiana were able to reproduce on *Rag2* soybean plants but were poorly adapted to *Rag1* soybean plants [26]. Most recently, biotype 4 aphids that can reproduce on both *Rag1* and *Rag 2* soybean plants were found in Wisconsin [27]. Cooper, et al. [23] studied the geographic distribution of the SBA biotypes across 11 states and one Canadian province between 2008 and 2010. The frequency of aphid populations belonging to biotypes 2, 3, and 4 was 54, 18, and 7%, respectively. The aphid populations from Wisconsin, the state where the SBA was first reported in the U.S. in 2000, showed higher virulence variability [23]. Additionally, Zhong, et al. [28] reported at least four biotypes of SBA in China. These biotypes were named China Biotype 1 (virulence on host plants with *Rag5* or *Rag6*), China Biotype 2 (virulence on host plants with *Rag1*, *Rag3*, or *Rag5*), China Biotype 3 (virulence on host plants with *Rag1*, *Rag3*, or *Rag6*), and China Biotype 4 (virulence on host plants with *Rag1*, *Rag2*, *Rag3*, or *Rag5* genes) [28].

### 2.3. Aphid Effectors are Host-Specific and Undergo Selection Pressure

The SBA uses two saliva types, gelling and watery, when feeding. Aphids inject gelling saliva during the early stages of feeding to form sheaths around their stylets [29] and later inject watery saliva containing effector molecules into mesophyll or phloem cells [30]. Since effector molecules allow aphids to modulate the immune reactions of host plants, they are subject to the scrutiny of host defense mechanisms and undergo natural selection [31]. Such selection helps effectors evade the host defense system, maintain their virulence, and evolve new functions [32].

Transcriptomic and proteomic studies of the pea aphid (*Acyrthosiphon pisum* Harris) found many enriched salivary proteins undergoing positive selection [33]. Aphid effectors are host specific and target specific host proteins to induce susceptibility [34,35]. Rodriguez, et al. [34] reported that Mp1, an effector molecule produced by the green peach aphid (*Myzus persicae* Sulzer), specifically targets Vacuolar Protein Sorting-Associated Protein 52 (VPS52) proteins in the green peach aphid’s preferred hosts, but this interaction did not occur in the aphid’s poor-hosts. Furthermore, the reproduction of the green peach aphid did not increase in *Arabidopsis* that expressed orthologs of the pea aphid’s effectors, including C002, PIntO1 (Mp1), and PIntO2 (Mp2) [35]. Since the identification and functional characterization of the first aphid effector molecule, C002 in the pea aphid [36], a wide range of effector molecules have been identified from different aphids. The availability of the whole genome sequences of several aphid species, including the pea aphid [37], the Russian wheat aphid (*Diuraphis noxia* Kurdjumov) [38], the green peach aphid [39], and the SBA [40], has facilitated the study of aphid salivary effector gene families. Carolan, et al. [41] identified 324 secretory proteins in the salivary glands of the pea aphid. Some, including glucose dehydrogenase, glutathione peroxidase, putative sheath protein of aphids, and angiotensin-converting enzyme-like, showed similarity to known aphid effectors [42,43,44], while others were more similar to nematode effectors, including M1 zinc metalloprotease, disulfide isomerase, calreticulin, Armet, glutathione peroxidase, and CLIP-domain serine protease [41,45,46]. Boulain et al. [33] identified 3603 candidate effector genes predicted to be expressed in pea aphid salivary glands and found that 740 of those were up-regulated in salivary glands [33]. Thirty-four salivary genes were identified in the Russian wheat aphid that were similar to the most commonly expressed genes in other aphids [38]. An intensive analysis of the genome of the green peach aphid, which can infest plant species belonging to 40 families, demonstrated the role multigene clusters play in allowing the species to colonize distantly related plant species [39]. The authors suggested genes belonging to the cathepsin B and RR-2 cuticular protein gene families undergo rapid transcriptional plasticity, and that this allows the green peach aphid to infest a wide range of plant species.

RNA-sequencing (RNA-seq) has become a standard tool for studying qualitative and quantitative gene expression [47,48]. Bansal et al. [49] studied xenobiotic stress response in SBA using RNA-seq. The authors found 914 significantly expressed genes in the SBA, most of which were related to stress and detoxification, including cytochrome p450s (CYPs), glutathione-S-transferases, carboxyesterases, and ABC transporters. Wenger, et al. [40] identified 135 putative SBA effector genes, including 68 CYP protein-coding genes (detoxification genes), 82 genes belonging to ABC transporter subfamilies, 14 glutathione-S transferases, and 17 carboxyl and choline esterases. The detoxification genes help SBA adapt to host plants [49]. The small number of CYP genes found in the SBA, the pea aphid (83 CYP genes), and the Russian wheat aphid (48 CYP genes) may explain why these species are adapted to a limited range of hosts, while the green peach aphid (115 CYP genes) is adapted to a wide range of hosts [50]. The availability of genome sequences for the SBA might be used to explain the species’ rapid adaptation to resistant soybean cultivars despite the lack of both genetic differentiation and selection pressure between avirulent and virulent biotypes [51].

### 2.4. Soybean Cultivars Exhibiting Antibiosis, Antixenosis, and Tolerance as A Resistance Response to Soybean Aphids

Smith 1989, 2005 [52,53] grouped plant resistance mechanisms to insects into three categories: antibiosis, antixenosis, and tolerance. Antibiosis resistance affects the biology, including the mortality or fecundity, of the insect. The soybean cultivar ‘Dowling’ exhibits antibiosis, and resistance factors are present in the phloem cells [54]. Antixenosis resistance affects the behavior of the insect. The soybean cultivar PI200538 exhibits both antibiosis and antixenosis [9,54]. Jesus, et al. [55] studied the physiological responses of 14 soybean genotypes to aphid infestation in terms of total protein, peroxidase level, and chlorophyll content. The genotypes UX 2569-1592-01 (*Rag2* gene; PI243540) and UX 2570-171-04 showed high and moderate levels of antibiosis and/or antixenosis, respectively. Chlorophyll content was unaffected except in UX 2569-159-2-01, which exhibited reduced chlorophyll content at 5 and 15 days after infestation. Total protein content remained unchanged between the infested and control plants. Tolerance resistance is the ability of the plant to endure the presence of the insect without affecting the pest’s biology or behavior [56]. The KS4202 cultivar is tolerant of aphids [57]. The tolerance effect in KS4202 may be attributable to the quick regulation of RuBP (ribulose-1,5-biphosphate) and the upregulation of detoxification genes [58].

### 2.5. Rag Genes in Soybean Cultivars Provide Resistance to Soybean Aphid

*Rag* (resistance to *Aphis glycines*) loci were first discovered in Dowling, PI71506, and Jackson cultivars [59] and have since been identified in other soybean lines. The mapping and inheritance mechanism of the *Rag1* gene has been documented in multiple soybean cultivars [60,61,62,63]. *Rag1* loci were mapped as a 115 kb interval on chromosome 7 using the Dowling (PI548663; donor parent of *Rag1*) and Dwight (PI587386; aphid-susceptible parent) cultivars [64]. *Rag2* loci were finely mapped as a 54 kb interval on chromosome 13 in the antixenotic PI200538 cultivar [54,65]. *Rag3* loci were mapped on chromosome 16 (LG J) using PI567543C [66]. The recessive *rag4* loci were mapped on chromosome 13 (LG F) between markers Satt649 and Satt343 (1,225,665–16,340,514 bp) in PI567541B [67]. The authors also mapped *rag1* provisional (*rag1c*) in chromosome 7 (LG M). *Rag5* (proposed) and *Rag6* have been identified in PI567301B and PI567598B, respectively [68,69]. Bhusal, et al. [70] identified two major and two minor loci. The major loci were located on chromosome 7 (*qChrom.07.1*) (1Mb distant from *Rag1*) and chromosome 16 (*qChrom.16.1*) (near *Rag3*); the minor loci were located on chromosome 13 (*qChrom.13.1*) (near *Rag4*) and chromosome 17 (*qChrom.17.1*) and were associated with aphid resistance in PI603712. Hill, et al. [71] characterized multiple SBA biotype resistances in five cultivars.

More than half of the genetic diversity has been lost in the cultivated soybean [72], but its closest wild relative, *Glycine soja* Siebold & Zucc., may be useful for identifying aphid-resistance genes, studying inheritance patterns, and mapping important resistance loci [24]. Hesler and Tilmon [73] reported PI135624 and PI65549 to be resistant to aphids, and Conzemius [74] reported that PI101404A and PI65549 were highly resistance to biotype 4 colonies. *Rag6* and *Rag3c* were mapped in 49-kb (42,146,252–42,195,720 bp) and 150-kb intervals (6,621,540–6,771,675 bp) on chromosome 8 and chromosome 16, respectively, in *G. soja* [75]. The 49–kb interval where *Rag6* was mapped contained three clustered Nucleotide Binding Site—Leucine-Rich Repeat (NBS–LRR) genes (*Glyma.08g303500*, *Glyma.08g303600*, and *Glyma.08g303700*) and one amine oxidase gene (*Glyma.08g303800*). The 150-kb interval where *Rag3c* was mapped contained one LRR gene (*Glyma.16g066800*) and another ten genes belonging to lipase, cytochrome P450, methyltransferases, hydrolases, and Ku70-binding gene families. All identified *Rag* QTLs in various soybean plant introductions (PI) are presented in Table 1.

**Figure 2:**
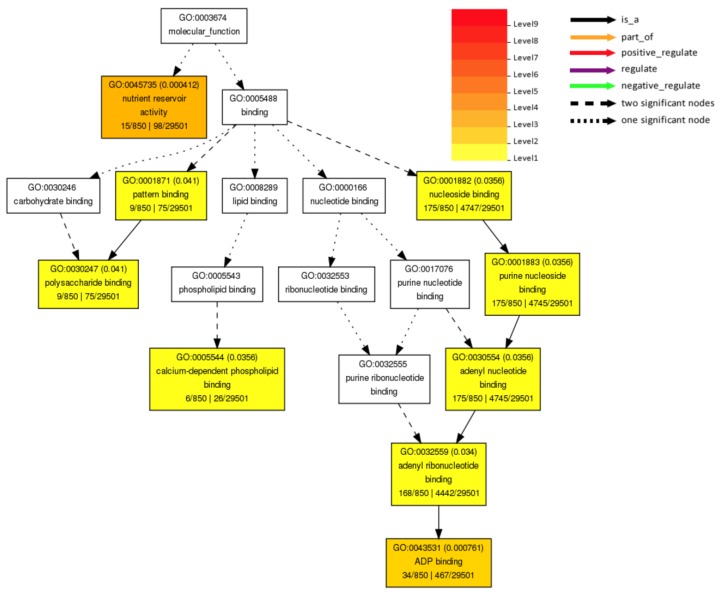
Significantly enriched gene ontology (GO) molecular function terms of 1,691 non-redundant genes in the *Rag* QTLs, including *Rag1* [64], *rag1b* [69], *rag1c* [67], *Rag2* [76], *Rag3* [66,77], *Rag4* [77], *rag3* [69], *rag3b* [78], *Rag3c* [75], *rag4* [67], *Rag5* [68], *Rag6* [75], as well as *qChrom.07.1*, *qChrom.16.1*, *qChrom.13.1*, and *qChrom.17.1* [70], as determined by Fisher’s exact test using AgriGO [80]. The same gene can be associated with multiple GO annotations. Only significantly (*p* < 0.05) over-represented GO categories are shown. The stronger colors (red and orange) represent lower *p*-values. Each box consists of the following information: GO term, adjusted *p*-value, GO description, number of query list and background mapping GO, and total number of query list and background.

Over 1600 non-redundant genes assessed from the *Rag* QTLs, including *Rag1* [64], *rag1b* [69], *rag1c* [67], *Rag2* [76], *Rag3* [66,77], *Rag4* [77], *rag3* [69], *rag3b* [78], *Rag3c* [75], *rag4* [67], *Rag5* [68], and *Rag6* [75], as well as *qChrom.07.1*, *qChrom.16.1*, *qChrom.13.1*, and *qChrom.17.1* [70], are significantly associated with nutrient reservoir activity (GO: 0045735) and binding (GO: 0005488). The “nutrient reservoir activity” molecular function is important in protecting plant tissues that produce surface waxes [79]. Similarly, the “binding” molecular function indicates that these genes play an important role in signaling and stress responses. The genes engaged in the process of binding (GO: 0005488) belong to adenosine diphosphate (ADP) binding (GO: 0043531), adenyl ribonucleotide binding (GO: 0032559), calcium-dependent phospholipid binding (GO: 0005544), adenyl nucleotide binding (GO: 0030554), purine nucleoside binding (GO: 0001883), nucleoside binding (GO: 0001882), pattern binding (GO: 0001871), and polysaccharide binding (GO: 0030247) gene families (Figure 2).

### 2.6. GWAS Studies on Sba Resistance on a Soybean Expanding Number of QTLs

Genome-Wide Association Studies (GWAS) have been an important alternative to classical bi-parental QTL mapping [82] for understanding the genetic basis of diseases linked to polygenic traits. The capacity of classical QTL mapping to identify allelic diversity and resolve genomes is limited [83], but GWAS can capture all recombination events undergone during the evolution of sampled genotypes [84]. Different kinds of phenotypes, including quantitative, binary, and ordinal phenotypes, can be studied using GWAS [85], and these phenotypes can be correlated with genotypes using mixed linear models [86]. Chang and Hartman [87] reported the first GWAS study for aphid-resistance using United States Department of Agriculture (USDA) soybean germplasms. The authors suggested that ss715596142 may be a significant Single Nucleotide Polymorphism and found three LRR domain containing genes (*Glyma07g13440*, *Glyma07g14810*, and *Glyma07g14791*) and one MYB transcription factor (*Glyma07g14480*). This marker is close to the *rag1c* gene that was reported in PI567541B [67], but it is not close to *Rag1* gene that contains the candidate LRR genes (*Glyma07g06890* and *Glyma07g06920*) [64]. More recently, Hanson, et al. [81] reported a significant number of SNPs on chromosomes 7, 8, 13, and 16, where *Rag* genes have been previously mapped, for multiple aphid biotypes, and also reported markers on chromosomes 1–2, 4–6, 9–11, 12, 14, and 16–20, where *Rag* genes had not been previously reported.

### 2.7. Rag Gene Pyramiding Provides Resistance to All Soybean Aphid Biotypes

Virulence in *Rag* soybean cultivars imposes a fitness cost on soybean aphids, and this could be used to preserve the efficacy of resistance genes in those cultivars [88,89]. In addition, the use of susceptible soybean plants as refuges for avirulent aphids might limit the frequency of virulent biotypes [88]. Soybean aphids are more virulent in cultivars with a single *Rag* gene than those with pyramided genes [52], and the pyramiding of resistance genes protects plants from multiple aphid biotypes [90,91]. The first soybean cultivar with both *Rag1* and *Rag2* genes became commercially available in 2012 and was resistant to aphid biotypes 2 and 3 [92]. Further pyramiding of *Rag1*, *Rag2*, and *Rag3* resistance genes may provide comprehensive resistance to all known aphid biotypes [89,91].

### 2.8. Transcriptomic Studies on Soybean–SBA Interaction: Jasmonic Acid (JA) and Abscisic Acid (ABA) Signaling Pathways Play a Crucial Role in Plant Resistance

Several studies have described the differential changes in phytohormones that occur during aphid-feeding in resistant, tolerant, and susceptible cultivars [93,94,95,96,97]. Different markers and responsive genes for salicylic acid (SA) are expressed cyclically in aphid-infested plants, indicating that SA may play a role in soybean resistance to aphid feeding [94]. Furthermore, the application of methyl jasmonate (MeJA) to infested plants significantly decreased SBA populations, but similar salicylic acid applications did not; this suggests MeJA may be an elicitor that induces plant defenses [94]. Thus, the JA signaling pathway, which functions in initiating the production of other enzymes, including polyphenol oxidase (PPO), lipoxygenases, peroxidases, and proteinase inhibitors, appears to play a crucial role in SBA resistance [94,98]. 

Brechenmacher et al. [56] used two *Rag2* and/or *rag2* near-isogenic lines of soybean to identify 396 proteins and 2361 genes that were differentially regulated in response to SBA infestation. Several genes mapped within the *Rag2* locus, including a gene of unknown function (*Glyma13g25990*), a mitochondrial protease (*Glyma13g26010*), and a NBS-LRR (*Glyma13g25970*), were significantly upregulated in the presence of aphids. Prochaska et al. [57] identified 3 and 36 differentially expressed genes (DEGs) at 5 and 15 days after infestation, respectively, in the resistant (tolerant) KS4202 cultivar but found only 0 and 11 DEGs at 5 and 15 days after infestation, respectively, in the susceptible K-03-4686 cultivar. Most of the DEGs were related to WRKY transcription factors (such as WRKY60), peroxidases (*Peroxidase 52* (*PRX52*) and *Ascorbate peroxidase 4* (*APX4*)), and cytochrome p450s. Aphid-tolerance mostly depended on the constitutive levels of abscisic acid (ABA) and jasmonic acid (JA) and the basal expression of ABA (*NAC19* and *SCOF-1)* and JA (*LOX10*, *LOX2* (a chloroplastic-like linoleate 13S-lipoxygenase 2), *OPDA-REDUCTASE 3* (*OPR3*)) related transcripts [93]. In addition, the genes *PRX52*, *WRKY60*, and *PATHOGENESIS-RELATED1* (PR1; SA-responsive transcript) were found to be induced by aphid infestation in the tolerant KS4202 cultivar [93]. Lee, et al. [99] evaluated the transcriptomic dynamics of soybean near-isogenic lines (NILs) with the *Rag5* or *rag5* alleles for resistance or susceptibility, respectively, to SBA biotype 2. Three genes located near the *Rag5* locus, including *Glyma.13 g190200*, *Glyma.13 g190500*, and *Glyma.13g190600*, were reported to be strong candidate genes for imparting SBA resistance. Li et al. [96] studied soybean responses to aphid infestation by using complementary DNA (cDNA) microarrays to generate transcript profiles and identified 140 genes related to the cell wall, transcription factors, signaling, and secondary metabolism. Studham and MacIntosh [97] utilized oligonucleotide microarrays to study soybean-SBA interactions in the aphid-resistant (*Rag1*) cultivar LD16060 and the aphid-susceptible cultivar SD01-76R. They identified 49 and 284 differentially expressed genes (DEGs) at 1 and 7 days after infestation, respectively, in the susceptible cultivar and found only 0 and 1 DEGs at 1 and 7 days after infestation, respectively, in the resistant cultivar. They suggested that the expression of defense genes in resistant plants is constitutive, whereas the defense genes in susceptible plants are expressed only after aphid infestation. A recent study by Hohenstein, et al. [100], compared the responses of resistant (*Rag1*) and susceptible plants after they were colonized by aphids for 21 days. They found that resistant plants exhibited a reduced response, while susceptible plants exhibited a strong response characterized by upregulation of genes involved in chitin regulation and isoflavonoid synthesis.

## 3. Soybean Cyst Nematode 

### 3.1. The Relationship between SCN and Soybean

SCN is an obligate, sedentary endoparasite that completes its life cycle in three to four weeks [101]. Organic molecules secreted by host plants signal key events, including egg hatching and second-stage juvenile (J2) dispersal, in the nematode life cycle. In soybean these molecules include eclepsins and glycinoeclepin A [101,102,103]. Other compounds, such as solanoeclepin A, picloronic acid, sodium thiocyanate, alpha-solanine, and alpha-chaconine, have also been found to initiate the egg hatching process in most nematodes [104,105]. *Gro-nep-1* has been recently identified as the first gene to be upregulated in eggs treated with host root exudate in golden nematode (*Globodora rostochiensis* Wollenweber) [106]. The exudates are used by the J2 nematodes to find the host plant’s root system [107,108], and nematodes that fail to enter a host plant die of starvation [109]. Once a J2 nematode locates a host, it infects the root cells using its stylet and secretes digestive enzymes, such as cellulase, to facilitate its movement through epidermal and cortical cells towards a vascular cylinder [107,110]. At the vascular cylinder, a J2 nematode induces a single cell to undergo morphological changes in order to form a permanent feeding site called a syncytium [107,110]. The syncytium remains intact throughout the remainder of the nematode’s life cycle [107]. The nematode then molts into the third juvenile stage (J3) and undergoes sexual differentiation [111]. The ratio of female to male J3 nematodes is generally one-to-one but is sometimes affected by the milieu and resistance of the host plant [112]. The feeding site swells longitudinally throughout the root as it dissolves and incorporates numerous cells with dense cytoplasm, hypertrophied nuclei, and increased organelle content [107]. The J3 male metamorphoses to a vermiform-shape, leaves the root to locate females, and dies after mating [113,114]. Concurrently, the J3 female molts to form an adult female which changes into a lemon-shaped cyst that extrudes from the root surface. Each female produces between 40 and 600 eggs with an average of approximately 200 eggs; eggs are occasionaly produced outside the cyst in adjacent gelatinous secretions [115,116]. Cysts produce compounds such as chitinase and polyphenol oxidase to protect eggs from desiccation and microbial infection [7] and can remain viable for up to nine years [7].

### 3.2. SCN Effectors Interact with Host Proteins and Enhance Either Susceptibility or Resistance in the Host Plants

Nematode effector molecules are produced in a nematode’s esophageal gland before being released into the stylet [117]. The effectors evade and suppress the host plant’s defense and reprogram the host cell nucleus, as well as a various cellular process, to facilitate invasion [118,119]. These effector molecules use various proteins, including cellulose binding proteins and expansins, to dissolve the cell wall and penetrate the host cell [118]. Nematodes parasitize soybeans by interacting with the host plant’s immune regulators, modifying its cell walls, mimicking plant hormones, and manipulating hormone transport [reviewed in [120]]. Various nematode effector molecules, including Gr-SPRYSEC (-4, -5, -8, -15, -18, -19), Gp-RBP-1, Gr-VAP1, Hg30C02, Hs10A06, Hs4F01, and Mi-CRT, have already been characterized in different nematodes and hosts [121,122,123,124,125,126,127,128]. These effectors affect the host immune system by enhancing susceptibility or resistance.

The characteristic cyst nematode effectors, including those found in SCN, are presented in Table 2. Gao, et al. [129] identified 51 effector molecules from the esophageal gland of the SCN. Most of the effector molecules were attributable to cellulose genes, pectate lyases, an enzyme in the shikimate pathway, and ubiquitin proteins. The ortholog of *H. glycines* cellulose binding protein (HgCBP) in *H. schachtii* (HsCBP) interacts with the pectin methyltransferase protein (PME3) of *Arabidopsis* during the early feeding stage and induces enhanced susceptibility [130]. Pogorelko et al. [131] studied the function of an ortholog of the 25A01-like effector family in *H. schachtii* (Hs25A01) in *Arabidopsis*. Hs25A01 interacts with *Arabidopsis* F-box-containing protein, chalcone synthase, and the translation initiation factor eIF-2 b subunit to increase both root length and susceptibility to *H. schachtii*. Pogorelko et al. also reported 18 more effector molecules that were similar to N-acetyltransferases, β-fructofuranosidases, serine proteases, cysteine proteases, an effector for protein degradation in the syncytium, cellulose binding protein, chorismate mutase, and glycosyl hydrolase. Among them, HgGLAND18, which is secreted in the dorsal gland cell, suppresses innate immune responses in *Nicotiana benthamiana* [132]. The similarity of the N-terminal domain of HgGLAND18 to the same domain of an effector in *Plasmodium spp.* suggests that convergent evolution has occurred in the effector molecules of diverse parasites [132]. Another effector, biotin synthase (HgBioB), and a protein containing a soluble N-ethylmale-imide-sensitive factor-attachment protein receptors (SNARE) domain (HgSLP-1) were recently reported using an allelic imbalance analysis [133]. HgSLP-1 interacts with Rhg1 soluble N-ethylmaleimide–sensitive factor attachment protein (α-SNAP) to evade its host’s defense [133]. However, *H. glycines* also produces a map-1 protein and Mj-Cg-1 effectors that allow it to evade host defenses in the absence of HgSLP-1 [133,134,135]. The use of transcriptomics has greatly expanded the number of putative effectors known from SCN. Gardner et al. [136] used a joint pipeline that utilized the presence or absence of signal peptides to predict 944 total effector candidates in the second stage juvenile *H. glycines*; many of these were homologs to glutathione synthetase, C-type lectins, plants RING/U-box superfamily, arabinosidase, fructosidase, glycoside hydrolase, expansin, and SPRYSEC family.

### 3.3. Rhg1 and Rhg4 as Major QTLs for SCN Resistance

SCN is capable of entering the roots of both susceptible and resistant soybean cultivars [141]. Resistant cultivars prevent SCN infection by disrupting syncytium formation. Histological experiments have determined that syncytia formation in resistant plants triggers a hypersensitive-like response [142]. The sources for SCN resistance in commercial soybean cultivars are predominantly Peking (PI548402), PI88788, and PI437654 [143,144] (Table 3). To date, 40 QTLs have been reported in a diverse group of resistant cultivars and have been mapped in 17 of 20 chromosomes [144]. Three recessive resistance genes, *rhg1*-*rhg3*, were initially identified in the Peking cultivar [145]. *rhg1* confers resistance to SCN in all germplasms with resistance to SCN and is a significant SCN resistance gene in soybean cultivars [144]. Moreover, PI437654 and PI88788 each have a different functional SCN resistance allele at or close to *rhg1* [143]. *rhg1* was initially reported as a recessive locus, but recent studies have shown that it exhibits incomplete dominance [146]. The *rhg1* locus has been present in various resistant plant introductions, including PI209332, PI437654, PI90763, PI209332, PI89772, PI90763, Peking (PI548402), PI88788, and PI437654 [144]. The *Rhg1* locus has been mapped to chromosome 18’s subtelomeric region [147,148,149,150]. *Rhg4,* a dominant locus, is present in PI54840 (Peking) and PI437654 but not in PI88788 or PI209332 [143,144,151]. The *Rhg4* locus has been mapped to chromosome 8 (linkage group A2) for SCN resistance [144,152].

### 3.4. Leucine-Rich Repeat Transmembrane Receptor-Like Kinase (LRR-RLK) Genes Were Considered as the Resistance Genes against SCN until 2010

The *Rhg1* and *Rhg4* genomic regions of the soybean and two leucine-rich repeat transmembrane receptor-like kinase (LRR-RLK) genes were patented as SCN resistance genes by two groups [147,148,166,167]. These claims were based on the similarity of the genes to the rice bacterial blight resistance gene *Xa21* [168]. The functional aspect of these claims were not studied until 2010. Melito, et al. [146] used artificial microRNA (amiRNA) to study the function of the *Glyma18g02680.1* gene (LRR-RLK) at the *Rhg1* locus. Reduced expression of *Glyma18g02680.1* did not alter plant resistance to SCN but instead affected root development. Later Liu, et al. [169] used the Targeting Induced Local Lesions In Genomes (TILLING) approach to study the function of LRR-RLKs at the *Rhg4* locus of developing EMS-mutants from the SCN-resistant soybean cultivars Forrest and Essex. They concluded that the *Rhg4 LRR-RLK* gene is not a gene for SCN resistance. The availability of the complete soybean genome has made it easier to narrow down these loci regions and find candidate genes for SCN resistance [170].

### 3.5. Roles of GmSNAP18 (Rhg1) and GmSHMT08 (Rhg4) in SCN Resistance

Kim, et al. [159] showed that *rhg1-b* was located within a 67-kb region in the PI88788 genotype. Because there are allelic variants of *rhg1* among different soybean genotypes, the *rhg1* in PI88788 was named as *rhg1-b* [143,159]. This 67-kb interval from PI88788 does not include the LRR-RLK gene candidate for *rhg1* that was previously patented from the Peking cultivar. Matsye, et al. [171] studied the expression of genes within the 67 kb interval of the *rhg1-b* locus. An amino acid transporter (*Glyma18g02580*) and a soluble NSF attachment protein (α –SNAP; *Glyma18g02590*) were specifically expressed in syncytia during SCN defense in both Peking (PI548402) and PI88788 genotypes. The α–SNAP coding regions are identical in the resistant genotypes Peking (PI548402) and PI437654 but contain a differing number of single nucleotide polymorphisms (SNPs) in the Williams 82 (PI518671) genotype [172]. Later, in a 31-kilobase (kb) segment at *rhg1-b* loci, the genes *Glyma.18G022400* (formerly *Glyma18g02580*), *Glyma.18G022500* (formerly *Glyma18g02590*), and *Glyma.18G022700* (formerly *Glyma18g02610*), which encode an amino acid transporter, an α-SNAP (soluble N-ethylmaleimide–sensitive factor attachment protein) protein, and a WI12 (wound-inducible domain) protein, respectively, were determined to play a significant role in SCN resistance [173,174]. The WI12 protein may be involved in producing phenazine-like compounds, which can be toxic to nematodes [173,175]. α -SNAP protein is involved in vesicle trafficking and affects the exocytosis of food in syncytium, which in turn affects nematode physiology [173]. The plant transporter protein, *Glyma18g02580*, consists of tryptophan/tyrosine permease family domains [173]. Tryptophan is catabolized to form indole-3-acetic acid, which is a precursor of the hormone auxin [176]. This suggests that *Glyma18g02580* may affect auxin distribution in soybean [173]. The cultivars Peking-type and PI88788 type can be differentiated by selecting the *rhg1* resistance alleles of the *Glyma18g02590* (*GmSNAP18*) gene using two specific KASP (kompetitive allele-specific PCR) SNP markers [177]. The 31 kb segment is present as a single copy in the susceptible cultivar, while the resistant varieties PI88788 and Peking (PI548402) possess 10 and three tandem copies, respectively [173]. Additionally, Cook, et al. [178] tested *Rhg1* across 41 diverse soybean cultivars using whole genome sequencing (WGS) and fiber-FISH (fluorescence in situ hybridization) methods. That study identified seven *Rhg1* copies in PI548316, nine copies in PI88788, and 10 copies in PI209332, while the genomes of PI437654 and PI548402 (Peking), both of which show a high levels of SCN resistance, contained three copies of the *Rhg1* with the α-SNAP allele [178]. Lee, et al. [179] genotyped the *Rhg1* locus in 106 SCN-resistant *G. max* and *G. soja* genotypes by developing a genomic qPCR assay for identifying the copy number of the *Rhg1* locus and found 2–4, 6, 7, 9, and 10 copies in *G. max* and one three-copy variant in a *G. soja* genotype.

The use of forward genetics and functional genomics approaches showed that the Peking-type *rhg1* resistance in the Forrest cultivar depends on an SCN-resistant allele of the *Rhg4* (*GmSHMT08*) gene [180]. This kind of SCN resistance, which requires both *rhg1* and *Rhg4*, differs from the PI88788-type resistance, which requires only *rhg1* [152,180]. The SCN resistance allele of the *GmSHMT08* gene originated from a gene duplication event that occurred during the soybean domestication process [181]. A recent study by Liu, et al. [182] identified a ~14.3 kb interval at the *rhg1-a* locus of the Forrest cultivar that contains three genes and appears to confer resistance at that locus. These genes encode an armadillo/β-catenin-like repeat, an amino acid transporter, and a soluble N-ethylmelaimide sensitive factor (NSF) attachment protein (*GmSNAP18*). Genetic complementation analyses of *GmSNAP18* revealed that it functioned differently in PI88788-type *GmSNAP18* and Peking-type *GmSNAP18*. Thus Peking-type *GmSHMT08* (*Rhg4*) and Peking-type *GmSNAP18* (*Rhg1*) play different roles than PI88788-type *GmSHMT08* and PI88788-type *GmSNAP18*. Bayless, et al. [174] confirmed that resistant cultivars possess of a dysfunctional variant of resistance-type α-SNAP that impairs NSF protein function, reducing its interaction during 20S complex formation. This impairs vesicle trafficking and causes cytotoxic levels of NSF protein to accumulated in the syncytium. However, because of two duplication events that occurred 13 and 59 million years ago (mya) [170], soybean encodes an additional four α-SNAPs, including *GmSNAP02*, *GmSNAP09*, *GmSNAP11*, and *GmSNAP14*, which are known as wild-type α-SNAPs [174,183]. Among them, *GmSNAP11* is a minor contributor to SCN resistance, but *GmSNAP14* and *GmSNAP02* are not [183]. These wild-type α-SNAPs counteract the cytoxicity found in soybeans that carry haplotypes of *Rhg1* for SCN resistance [174]. In the presence of SCN, the ratio of resistance-type to wild-type α-SNAP increases and leads to the hyperaccumulation of resistance-type α-SNAP, which reduces the viability of the syncytium [174]. Also, the overexpression of additional genes, such as ascorbate peroxidase 2, β-1,4-endoglucanase, soybean momilactone A synthase-like, cytochrome b5, developmentally regulated plasma membrane polypeptides (DREPP) membrane protein-family, and plastocyanin–like including serine hydroxymethyltransferase, decreased the female index of SCN by 50% or more in the SCN susceptible cultivar William 82 [184].

Liu, et al. [185] used two recombinants with resistance alleles at the *rhg1* and *Rhg4* loci to study a gene at the *Rhg4* loci. The cultivars used in the study were double recombinants for an 8-kilobase (kb) interval carrying the *Rhg4* resistance allele that carries two important genes, serine hydroxymethyltransferase (SHMT) and the other a subtilisin-like protease (SUB1). The *SHMT* (*GmSHMT08* ) gene was confirmed as the resistance gene at the *Rhg4* locus. SHMT catalyzes methylene carbon of glycine to tetrahydrofolate (THF) to form methyleneTHF, which reacts the second glycine to form L-Ser in the glycolate pathway [186]. This reaction produces S-adenosyl-Met (SAM), which is the precursor for the polyamines and the plant hormone ethylene [180]. *GmSHMT08* changes the enzymatic properties of SHMT because of changes in two amino acids (P130R and N385Y) in the resistant allele. This negatively affects the folate homeostasis in the syncytium, resulting in hypersensitive responses (HR) leading to programmed cell death (PCD) [181,185]. The alleles of *GmSHMT08* are different between resistant and susceptible plants [185].

### 3.6. Minor QTLs/Genes for SCN Resistance

In addition to the major QTLs identified at *Rhg1* and *Rhg4* loci, there are minor SCN resistance genes or QTLs, such as *qSCN10* on chromosome 10 in PI567516C cultivar [164]. The PI567516C cultivar lacks the two major loci *Rhg1* and *Rhg4* and is SCN resistant; this implies that minor genes may confer SCN resistance [187]. The resistance conferred by the major genes is sometimes not durable and necessitates the use of horizontal or quantitative resistance acquired from minor genes [188]. Other minor QTLs are *qSCN-003* in PI88788 [160], *qSCN-005* in Hartwig, which has SCN resistance from PI437654 and Peking [161], and *qSCN-11* in PI437654 and PI90763 [156,165]. The most recently reported QTLs are cqSCN-006 and cqSCN-007 in *Glycine soja* PI468916 [162]. These were mapped finely by Yu and Diers [163], who mapped *cqSCN-006* to a 212.1 kb interval and *cqSCN-007* to a 103.2 kb interval on chromosomes 15 and 18, respectively, of the Williams 82 reference genome. The *cqSCN-006* QTL consists of three major candidate genes: *Glyma.15g191200* (Soluble NSF attachment protein), *Glyma.15g191300* (BED-zinc finger related), and *Glyma.15g191400* (BED-zinc finger related). *Glyma.15g191200* is predicted to encode a soluble N-ethylmaleimide–sensitive factor attachment protein (γ-SNAP) that possesses the same function as α-SNAP, which is one of the important genes in *Rhg1* mediated SCN resistance. Likewise, the potential genes identified in the region of *cqSCN-007* are: *Glyma.18g244500* (Lecithin-cholesterol acyltransferase), *Glyma.18g244600* (Apetala 2 transcription factor), *Glyma.18g244700* (Calcineurin-like phosphoesterase), *Glyma.18g244800* (Chromatin assembly factor 1 subunit A), *Glyma.18g244900* (p-Nitrophenyl phosphatase), *Glyma.18g245000* (Rad21/Rec8-like protein), and *Glyma.18g245200* (LETM1-like protein). These genes are mainly involved in signaling pathways, such as transcription, euchromatin expression, and membrane receptor detection. These identified genes might be novel SCN resistance genes and should be functionally characterized in the future [163].

### 3.7. GWAS Study in SCN Resistance Expands other QTLs on SCN

The GWAS technique has been used to identify candidate genes for SCN resistance in relatively less time while simultaneously verifying QTLs identified by classical bi-parental mating [82,83,84,189,190,191,192]. Wen, et al. [190] reported 13 GWAS QTLs for SCN resistance that were associated with the sudden death syndrome (SDS) QTLs; these spanned a physical region of 1.2 Mb (1.2–2.4 Mb) around three *Rhg1* genes. This might explain the close linkage of *Rfs2* and *Rhg1* genes that provide resistance to SDS and SCN, respectively [193]. Han, et al. [192] reported 19 significant QTLs related to resistance to both SCN HG Type 0 (race 3) and HG Type 1.2.3.5.7 (race 4) among 440 soybean cultivars. Of the reported SNPs, eight corresponded to QTLs with *Rhg1* and *Rhg4* genes, eight to other known QTLs, and three were novel QTLs located on chromosomes 2 and 20. The gene, *Glyma.02g161600*, which encodes the RING-H2 finger domain nearest to the novel loci, could be a new source of SCN resistance. Vuong, et al. [83] utilized 553 soybean Plant Introductions (PIs) and the SoySNP50K iSelect BeadChip (with 45,000 SNP markers) to detect the QTLs or genes for HG Type 0 SCN resistance. Fourteen loci with 60 SNPs were significantly associated with SCN resistance. Of the 14 detected loci, six QTLs that had been identified using bi-parental mapping, including *Rhg1* and *Rhg4*, were also verified. These GWAS QTLs contained 161 candidate genes located at significant GWAS loci for SCN resistance in soybean. Among them, 26 were NBS genes that encoded PF90031 domains. Chang, et al. [84] reported significant loci for resistance to multiple races of SCN, including one SNP that was within the *Rhg1* locus for SCN races 1, 3, and 5. Among the five LRR-RLK genes, *Glyma18g02681* and *Glyma20g33531* were nearest to two significant SNPs, s715629308 and ss715638409, respectively, and significant SNPs were reported to be located on chromosomes 4, 7, 10, 15, 18, and 19 for SCN races 1 and 5 (HG type 2). However, Li, et al. [189] employed joint linkage mapping and association mapping using 585 informative SNPs across recombinant inbred lines (RILs) bred from the cross Zhongpin03-5373 (ZP; resistant to SCN) × Zhonghuang13 (ZH; susceptible to SCN) to detect alleles associated with SCN race 3. Association mapping revealed three quantitative trait nucleotides (QTNs): *Glyma18g02590* (belonged to locus *rhg1-b*), *Glyma11g35820*, and *Glyma11g35810* (a *rhg1-b* paralog). Linkage mapping revealed two QTLs, including one mapping to *rhg1-b* and another to a *rhg1-b* paralog. Upon combining both linkage and association mapping, six significant markers were detected. Among them, four (Map-5118, Map-5255, Map-5431, and Map-5432) of the significant markers were not identified in the independent study. Map-5431 lies between *rhg1-a* and *rhg1-b* (*Glyma18g02650*), and Map-5432 lies adjacent to *rhg1-a* (*Glyma1802690*) [193].

Zhang, et al. [191] utilized 235 wild soybean (*G. soja* Sieb. & Zucc.) accessions to unravel the genetic basis for resistance to HG Type 2.5.7 (race 5). GWAS revealed 10 significant SNPs associated with SCN resistance, and four of these were linked to a known QTL, *rhg1*, on chromosome 18. Another four were linked to a race 5 resistance QTL [194], and the remaining two were linked to a 35.5 to 37.8Mb region that overlaps some regions identified by Vuong et al. [83]. Additionally, 58 potential gene candidates were identified that belonged to genes encoding NBS-LRR proteins (*Glyma.18G078000*, *Glyma.18G077900*), Mitogen Activated Protein Kinase (MAPK) proteins (*Glyma.18G106800*) RLPs (*Glyma.18G193800*), a RING/U-box protein (*Glyma.18G063500*), and MYB family transcription factors (*Glyma.19G119300*). Recently, Zhang, et al. [82], used a GWAS to dissect the genetic basis for resistance to race 1. Ten significant SNPs were identified on chromosomes 2, 4, 9, 16, and 18, including two which were within previously identified QTLs (SCN 18-5 and SCN 19-4 in chromosome 4 [194], one within QTL SCN 37-2 [187]). This study strongly indicated that the *R* gene, *Glyma.18G102600*, may be a promising candidate gene for SCN resistance because of its location in a strong linkage disequilibrium block.

**Figure 3:**
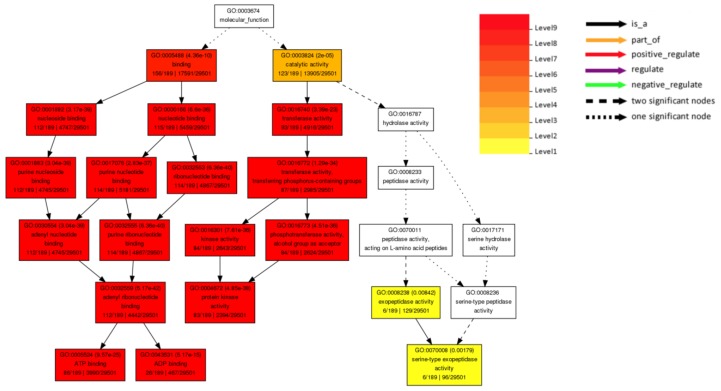
Significantly enriched gene ontology (GO) molecular function terms of 249 non-redundant genes in the GWAS SCN QTLs [82,83,84,189,190,191,192] as determined by a hypergeometric test using AgriGO [80]. The same gene can be associated with multiple GO annotations. Significantly (*p* < 0.01) over-represented and Bonferroni adjusted GO categories are shown. The stronger colors (red and orange) represent lower *p*- values. Each box consists of the following information: GO term, adjusted *p*-value, GO description, a number of query list and background mapping GO, and the total number of query list and background.

The 249 non-redundant genes assessed from the GWAS SCN QTLs [82,83,84,189,190,191,192] showed most of the genes functioned in binding (GO: 0005488) and catalytic activity (GO: 0003824). The binding category includes nucleoside binding (GO: 0001882), nucleotide binding (GO: 0000166), purine ribonucleotide binding (GO: 0017076), purine nucleoside binding (GO: 0001883), ribonucleotide binding (GO: 0032553), adenyl nucleotide binding (GO: 0030554), adenyl ribonucleotide binding (GO: 0032559), ATP binding (GO: 0005524), and ADP binding (GO: 0043531). The catalytic category includes transferase activity (GO: 0016740), transferase activity-transferring phosphorus-containing groups (GO: 0016772), phosphotransferase activity- alcohol group as acceptor (GO: 0016773), kinase activity (GO: 0016301), protein kinase activity (GO: 0004672), exopeptidase activity (GO: 0008238), and serine-type exopeptidase activity (GO: 0070008) (Figure 3). 

### 3.8. Transcriptomic Studies of SCN Reveal a Complex Network of Genes for SCN Resistance

Hosseini and Matthews [195] used transcriptomic and regulatory analyses to investigate the effects of NH1–RHg (Race 3) and TN8 (Race 14) SCN populations on soybean roots (Peking cultivar) at 6 and 8 days after inoculation (DAI). The authors found that *β*–1, 4-glucanase, chalcone synthase, and superoxide dismutase, as well as genes for heat shock proteins (HSPs) and isoflavonoids, exhibited race-specific expression in the roots. Additionally, 30 of 46 transcription factor binding sites (TFBS), including HAHB4, MYB77, and OsCBT, were either over- or underrepresented in both races. Li, et al. [196] studied time course (5, 10, 15 DAI) transcriptomic changes in SCN-resistant and SCN-susceptible lines of soybean (ZDD2315-resistant, Liaodou15-susceptible) infected by SCN race 3. *Rhg1* and genes related to cytochrome P450, isoflavone-related pathways, phytoalexin synthesis, pathogen-related proteins, and transcription factors, including bHLH, MYB, LOB, bZIP, WRKY, C2H2, and NAC, were differentially expressed in these cultivars.

Recent research on the transcriptomics of SCN has been carried out in wild relatives of soybean or other hosts. Zhang, et al. [197] performed RNA-seq analysis in two different cultivars of *G. soja*, including a resistant genotype (PI424093) and a susceptible genotype (PI468396B), using SCN HG type 2.5.7. The number of differentially expressed genes in the resistant cultivar (2,290 genes) was higher than in the susceptible cultivar (555 genes) and included genes related to pathogen recognition, calcium-mediated defense, hormone signaling, MAPK signaling, and WRKY transcription factors. Interestingly, they found 16 NBS-LRR genes that showed significant expression upon SCN infection; among these was *Glyma.17G180000*, which was strongly induced in the PI424093 cultivar. Jain, et al. [198] studied the effect of SCN HG Type 0 in resistant (PI533561) and susceptible (GTS-900) cultivars of the common bean (*Phaseolus vulgaris*) 8 DAI. The authors reported a successful infection of SCN in the common bean for the first time. Various transcription factors (TFs), protein kinases, NBS encoding genes, WRKY transcription factors, pathogenesis-related (PR) proteins, and heat shock proteins were differentially expressed in interactions between common bean and SCN. A recent study by Tian, et al. [199] utilized small RNAs in a soybean-SCN interaction study. MicroRNAs (miRNAs) play a crucial role in regulating the transcription and translation of various genes [200]. The authors utilized susceptible (KS4607) and resistant (KS4313N) soybean cultivars and SCN HG type 7 to study the effects of soybean miRNAs during SCN infection. Both conserved (gma-miR159, gma-miR171, gma-miR398, gma-miR399, and gmamiR408) and legume-specific miRNAs (gma-miR1512, gma-miR2119, and gma-miR9750) were identified as potential candidates for the manipulation of SCN infection.

## 4. Aphid-Nematode Interactions in the Host Plant Reveal Communication via Systemic Tissues: Soybean–SBA–SCN Relationship

Infection of a plant by pests leads to a series of cell signaling events, including plasma membrane potential variation, calcium signaling, and generation of reactive oxygen species, which in turn lead to the production of hormones and metabolites [201]. In most cases, the release of hormones are specific to a corresponding stimulus. For example, jasmonic acid (JA) is produced in response to chewing herbivores, cell content feeders, and necrotrophic pathogens, while salicylic acid (SA) is produced in response to piercing-sucking herbivores [202]. However, ethylene (ET) is produced synergistically with JA and modulates both the JA and SA signaling pathway [203]. The change in metabolite products during herbivore feeding occurs in both local and systemic tissues [204]. Both above- and belowground herbivores, though segregated, share a host plant and influence each other [205]. The populations of numerous belowground organisms that feed on plant roots, such as nematodes, pathogens, fungi, and insects, can fluctuate in response to the concentration of plant defense compounds, such as phenolics, terpenoids or glucosinolates, which occur in both belowground and aboveground plant tissues [206]. The impact of root-feeders on shoot defense, and the effects of aboveground herbivory on root defense, has remained understudied [207], although many studies to understand relationship between plant-aphid-nematode interactions have been done [119,206,208,209,210,211,212,213,214,215,216,217,218,219,220] (Table 4). 

The interaction between insect herbivores and their hosts creates a condition called induced susceptibility, which assists subsequent herbivores [221], and this type of susceptibility occurs among conspecific herbivores on both susceptible and resistant plants [221,222]. The phenotypes of conspecifics can be either virulent and avirulent. For example, the survival of avirulent *Myzus persicae* (Sulzer) increased on resistant plants that were first fed on by avirulent *M. persicae* [223]. Hence, diverse populations containing both virulent and avirulent phenotypes can stimulate induced susceptibility on resistant plants [224]. Varenhorst, et al. [225] and Neupane, et al. [226] concluded that feeding by virulent soybean aphids increases the susceptibility of otherwise resistant soybean plants to avirulent conspecifics. Induced susceptibility arises two different ways in *A. glycines:* feeding facilitation and obviation of resistance [222,227]. Feeding facilitation refers to a condition in which conspecifics are favored on either susceptible or resistant host plants in the presence of another herbivore, irrespective of its genotype. Obviation of resistance refers to a condition in which feeding by virulent herbivores increases the susceptibility of resistant plants to avirulent conspecifics. The influence of SCN on SBA infestation or *vice versa* has been studied on soybean [211,212,220,228,229,230].

A study on the interaction effects of SCN and SBA on the ‘Williams’ soybean cultivar found that aphid populations were unaffected by SCN infection in laboratory conditions [211]. This study was validated in the natural field conditions, including both open plots and experimental cages, although aphids preferentially colonized soybean plants that were not infected by SCN. Heeren, et al. [229] utilized resistant and susceptible soybean lines with respect to both SBA and SCN in order to study the interaction effects of SBA and SCN in field conditions. The effect of SBA feeding on SCN reproduction was not observed in any of the soybean cultivars. McCarville, et al. [220] conducted experiments on different SCN susceptible and SCN resistant soybean cultivars to understand the effects of multiple pest/pathogen (SBA, SCN, and the fungus *Cadophora gregata*) interactions. The study showed that the SCN reproduction was increased (5.24 times) in the presence of SBA and *C. gregata*. In contrast, the aphid population decreased by 26.4% in the presence of SCN and *C. gregata*, and the SCN resistant cultivars (derived from PI88788) reduced aphid exposure by 19.8%. McCarville, et al. [212] demonstrated the relationship between aboveground SBA feeding and belowground SCN reproduction in SCN resistant and SCN susceptible soybean cultivars. In that experiment, SBA feeding improved the quality of soybean as a host for SCN, but the result varied significantly with both the cultivar type and the duration of the experiment. After 30-days, the number of SCN eggs and females increased by 33% (1.34 times) in the SCN-resistant cultivar and were reduced by 50% in the SCN-susceptible cultivar. After 60-days, the numbers of SCN eggs and females remained unaffected in the resistant cultivar but decreased in the susceptible cultivar.

## 5. *PHYTOALEXIN DEFICIENT4* (*PAD4*) is Involved in both SBA and SCN Interactions in Soybean

The *PHYTOALEXIN DEFICIENT4* (*PAD4*) gene encodes a lipase-like protein [231] and interacts with *ENHANCED DISEASE SUSCEPTIBILITY 1* (*EDS1*) and *SAG101* (*SENESCENCE ASSOCIATED GENE101*) [232,233] to promote the accumulation of salicylic acid in response to aphid infestation [234]. Extensive research on AtPAD4 has indicated that it functions in resistance to the green peach aphid, *M. persicae* [234,235,236,237,238]. The *PAD4* gene is expressed at the site of insect feeding and induces antibiotic and antixenotic defenses against aphids [234]. While the *PAD4* gene requires the co-occurrence of *EDS1* to provide resistance against bacteria and fungi, *PAD4* mediated resistance to *M. persicae* does not require *EDS1* [235,236]. However, the *PAD4* gene interacts with *SAG13*, *SAG21*, and *SAG27* genes to initiate premature senescence of *M. persicae* infested leaves as a form of basal resistance in *Arabidopsis* [235]. Although the function of the *PAD4* gene is widely studied in *M. persicae* and *Arabidopsis* system, there are few studies in the SBA and host soybean system. In resistant cultivars, such as *Rag1* cultivar Dowling, *GmPAD4,* a gene induced by the SBA, contributes to antibiosis [239]. Also, the high expression of a splice variant of *GmPAD4*, *GmPAD4-AS1*, in the *Rag1* Dowling cultivar suggests it functions in defense against aphid infestation [240].

**Figure 4:**
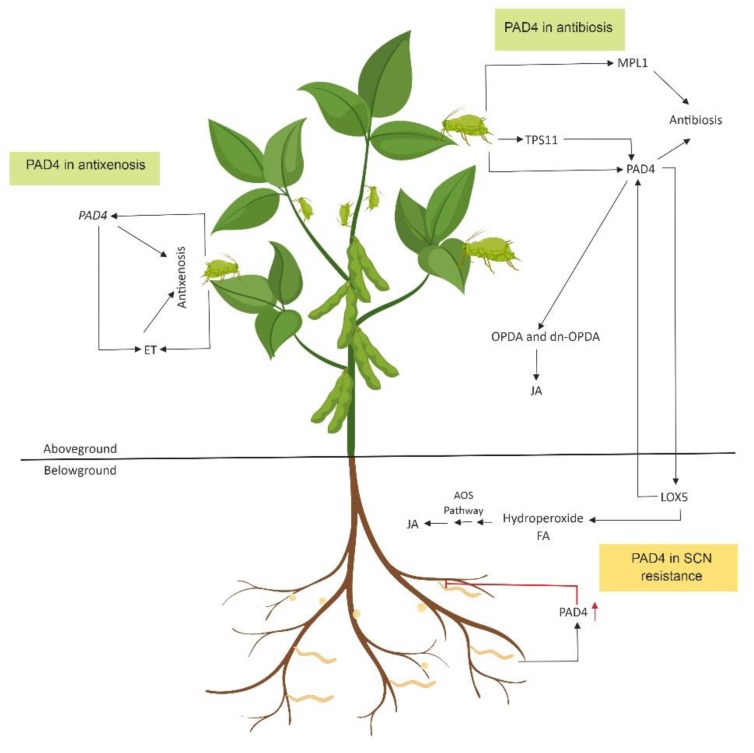
The role of *GmPAD4* in soybean aphid (SBA) and soybean cyst nematode (SCN) resistance. The pathways involved are adapted from several studies [234,235,236,237,239,240,241,242,243,244,245,246,247]. In the shoot, PAD4 is induced by SBA feeding and provides both antibiosis and antixenosis modes of resistance against the aphid. The function of PAD4 is well studied in both *M. persicae-Arabidopsis* and soybean-SBA systems [238,239]. The involvement of TPS11 and MPL1, both of which are regulators of PAD4, in aphid resistance (antibiosis) is well studied in the *M. persicae-Arabidopsis* system [253,254,255]. The antixenosis mode of resistance against the aphid is caused by the accumulation of ethylene. In the *M. persicae-Arabidopsis* system, aphid feeding causes the accumulation of LOX5, a crucial enzyme in the jasmonic acid pathway, in the root [243,244]. In addition, LOX5 upregulates PAD4 in the shoot, leading to the production of *cis*-(+)-12-oxo-phytodienoic acid (OPDA) and dinor-12-oxo-phytodienoic acid (dn-OPDA) [245]. In the root, expression of PAD4 causes a negative effect on SCN [241]. Altogether, these studies suggest PAD4 is a key protein in interactions among SBA and SCN. Abbreviations used include: TPS11 (*TREHALOSE-6-PHOSPHATE SYNTHASE 11*), MPL1 (*MYZUS PERSICAE-INDUCED LIPASE 1*) PAD4 (*PHYTOALEXIN DEFICIENT4*), FA (Fatty Acids), AOS (Allene Oxide Synthase), JA (Jasmonic Acid), ET (Ethylene), LOX5 (*LIPOXYGENASE 5*), OPDA (*Cis*-(+)-12-Oxo-Phytodienoic Acid), and dn-OPDA (Dinor-12-Oxo-Phytodienoic Acid). This illustration was created using Biorender (https://app.biorender.com/).

A study on the expression of a gene encoding AtPAD4 in soybean roots revealed that it had a negative effect on SCN populations [241]. This study also showed that AtPAD4 expression had no influence on the production of *GmEDS1* transcripts but significantly increased the production of *GmPR1* transcripts. The expression of *PR1* depends on the accumulation of SA and is downstream in the SA pathway [242]. The infestation by *M. persicae* has been demonstrated to cause the accumulation of transcripts of *LIPOXYGENASE 5* (*LOX5*), an important enzyme in the jasmonic acid pathway, in the roots [243,244]. *LOX5* also upregulates *PAD4* expression upon *M. persicae* infestation [244]. This leads to the production of *cis*-(+)-12-oxo-phytodienoic acid (OPDA) and dinor-12-oxo-phytodienoic acid (dn-OPDA) [245]. This system also provides *M. persicae* resistance in *Solanum lycopersicum* when *SlPAD4*, the *S. lycopersicum* homolog of *Arabidopsis PAD4*, is expressed [246]. A recent study has shown that the tolerant soybean cultivar KS4202 expresses *LOX2*, *LOX10*, and *OPDA-REDUCTASE 3* (*OPR3*) at higher constitutive levels, suggesting that lipooxygenases and OPDA function in soybean resistance to SBA [93]. The role of OPDA and dn-OPDA in nematode resistance has been studied in the *Arabidopsis* and root-knot nematode (*M. hapla*) system using plants with mutations in the JA-biosynthetic pathway [247]. Altogether, these studies suggest *PAD4* and enzymes involved in the JA pathway play a crucial role in plant defense against both aphids and nematodes. Expression of the *GmPAD4* gene and modulation of lipoxygenases and OPDA concentrations in the soybean plant may play a crucial role in resistance to aboveground SBA and belowground SCN. The role of *PAD4* in SBA and SCN resistance is shown in Figure 4.

## 6. Conclusions and Future Directions

Resistance to SBA and SCN is in each case mediated by several genes, including *Rag* genes for SBA and *Rhg* genes for SCN. While significant progress has been made towards identifying genes for SCN resistance, the genes responsible for SBA resistance remain largely obscure. The advent of sequencing technologies has made the soybean, SBA, and SCN genomes available. This should speed the discovery of specific effectors and host resistance components. The use of new gene editing tools, such as the Clustered Regularly Interspaced Short Palindromic Repeat (CRISPR)-Cas9 system, to produce mutant hosts will help identify the function of putative resistance genes. Since SBA and SCN co-exist in many soybean fields, it would be most valuable to target resistance mechanisms common to both pests. Expression of *GmPAD4*, lipoxygenases, and OPDA may provide resistance to both SBA and SCN.

## Figures and Tables

**Table 1:** List of soybean cultivars for mapping *Rag* genes with chromosome locations, associated markers, and types of resistance. (**^γ^** = The position of the markers are based on Glyma 2.0 as of [81]).

QTLs	Soybean Plant Introductions	Chromosome (Linkage Group)	Markers Associated (Location^γ^)	Type of Resistance	References
*Rag1*	PI548663	7 (M)	Satt435 and Satt463		[62]
	PI71506	7 (M)		Antixenosis	[63]
	PI548663	7 (M)	46169.7and 21A (5,529,532–5,770,718 bp)	Antibiosis	[64]
	PI548657	7 (M)	Satt435 andSatt463		
	PI587663	7 (M)	Satt567 and Satt245	Antibiosis	[71]
	PI587677	7 (M)	Satt540	Antibiosis	[71]
	PI587685	7 (M)	Satt540	Antibiosis	[71]
	PI594592	7 (M)	Satt540	Antibiosis	[71]
*rag1c*	PI567541B	7 (M)	sat229–satt435 (2,434,259–8,234,168 bp)		[67]
*rag1b*	PI567598B	7 (M)	Satt567 and Satt435 (5,523,128–5,909,485 bp)		[69]
*Rag2*	PI243540	13 (F)	Satt334 and Sct_033(28,415,888–30,739,587 bp)	Antibiosis	[76]
	PI200538	13 (F)	Satt510, Soyhsp176, Satt114, and Sct_033 (29,609,521– 31,802,676 bp)	Antibiosis	[65]
	PI587663, PI587685	13 (F)	Satt114, SNP2, Satt335		[71]
	PI587677	13 (F)	Satt335		[71]
	PI587972	13 (F)	Satt114, Satt510		[71]
	PI594592	13 (F)	Satt114		[71]
*Rag3*	PI567543C	16 (J)	Sat_339 and Satt414 (4,964,852–7,212,164 bp)	Antixenosis	[66]
	PI587663	16 (J)	Satt285	Antibiosis	[71]
	PI594592	16 (J)	Satt654	Antibiosis	[71]
	PI567543C	16 (J)	ss715625290 and ss715625308 (6,314,060–6,571,305 bp)		[77]
*rag3*	PI567598B	16 (J)	Satt285 and Satt414 (6,314,120–6,570,336 bp)		[69]
*rag3b*	PI567537	16 (J)	4,964,852–7,957,026 bp	Antibiosis	[78]
*Rag3c*	E12901	16 (J)	Gm16-3 and Gm16-5 (6,621,540–6,771,675 bp)	Antibiosis	[75]
*rag4*	PI567541B	13 (F)	Satt649–Satt343 (1,225,665–16,340,514 bp)	Antibiosis	[67]
	PI587677	13 (F)	Satt586		[71]
*Rag4*	PI567543C	13(F)	MSUSNP13-29-ss247923149 (13,691,537–13,626,971 bp)		[77]
*Rag5* Proposed	PI567301B	13	4 SSR markers (30,236,183–30,749,047 bp)	Antixenosis	[68]
*Rag6*	E12901	8	Gm08–15 and Gm08–17 (42,146,252–42,195,720 bp)	Antibiosis	[75]
*qChrom.07.1*	PI603712	7(M)	ss715598483–ss715598534 (6,444,246–6,819,959 bp)		[70]
*qChrom.16.1*	PI603712	16(J)	ss715625261–ss715625278 (6,105,250–6,222,257 bp)		[70]
*qChrom.13.1*	PI603712	13(F)	ss715613721–ss715617240 (13,691,537–13,626,971 bp)		[70]
*qChrom.17.1*	PI603712	17(D2)	ss715627556–ss715627637 (39,019,814–39,521,449 bp)		[70]

**Table 2:** List of characterized cyst nematode effectors in different plant systems with their targets and susceptibility/resistance effects.

SCN Effectors	Cyst Nematode	Targets	Host	Effect	References
Hg30C02	*H. schachtii*	β-1,3-endoglucanase	*Arabidopsis thaliana*	susceptibility	[122]
Hs10A06	*H. schachtii*	Spermidine Synthase2 (SPDS2)	*Arabidopsis thaliana*	susceptibility	[123]
Gr-VAP1	*G. rostochiensis*	apoplastic cysteine protease Rcr3pim	*Solanum lycopersicum*	programmed cell death	[126]
Gp-Rbp-1	*G. pallida*	Gpa2	*Nicotiana benthamiana*	hypersensitive response (HR)	[127]
Gr- SPRYSEC (4,5,8,15,18,19)	*G. rostochiensis*	NBS-LRR proteins	*Nicotiana benthamiana*	Suppress host defense	[128]
HsCBP	*Heterodera schachtii*	pectin methyltransferase protein (PME3)	*Arabidopsis thaliana*	susceptibility	[130]
Hs25A01	*H. schachtii*	F-box-containing protein, a chalcone synthase and the translation initiation factor eIF-2 b subunit (eIF-2bs)	*Arabidopsis thaliana*	susceptibility	[131]
HgGLAND18	*H. glycines*	-	*Nicotiana benthamiana*	suppresses both canonical basal and HR immune responses	[132]
HgSLP-1	*H. glycines*	*Rhg1* α-SNAP	*Glycine max*	avirulence protein	[133]
Hs19C07	*H. schachtii*	auxin influx transporter LAX3	*Arabidopsis thaliana*	susceptibility	[137]
Hs4D09	*H. schachtii*	14-3-3ε	*Arabidopsis thaliana*	resistance	[138]
Hs10A07	*H. schachtii*	interacting plant kinase (IPK) and IAA16 transcription factor	*Arabidopsis thaliana*	hypersusceptible	[139]
Hs30D08	*H. schachtii*	SMU2 (homolog of suppressor of mec-8 and unc-52 2)	*Nicotiana benthamiana*	susceptibility	[140]

**Table 3:** List of SCN resistance QTLs in soybean cultivars with chromosome location, associated markers, and corresponding SCN HG types or races.

QTLs	Chromosome and Markers Associated	SCN HG Type or Races	Soybean Plant Introductions	References
*cqSCN-001* (*Rhg1*)	18	Race 3	PI437654	[153]
		Race 1a, 3a, 3b, 1b, 6	PI209332	[154]
		Races 2, 3 and 5	PI90763	[155,156]
		Races 1, 3, and 6	PI88788	[155]
		Races 1, 2, were verified in Peking conditioning resistance to SCN 3, 5	PI89772	[157]
		Races 2, 3 and 5	PI404198A	[158]
*rhg1-b*	18	PA3 (HG type 7) and TN14 (HG type 1.2.5.7)	PI88788	[143]
	18; 67-kb region of the ‘Williams 82’ genome between BARCSOYSSR_18_0090 and BARCSOYSSR_18_0094	PA3, which originally had an HG type 0 phenotype	PI88788	[159]
*cqSCN-002* (*Rhg4*)	8	Race 3	Peking	[141,152]
		Race 3	PI437654	[153]
*cqSCN-003*	16	PA3 (HG type 7, race 3) and PA14 (HG type 1.3.5.6.7, race 14)	PI88788	[160]
*cqSCN-005*	17	HG Type 1.3 (race 14) and HG Type 1.2.5 (race 2)	Hartwig (PI437654 and Peking)	[161]
*cqSCN-006*	15; (803.4 kb region between SSR markers BARCSOYSSR_15_0886 And BARCSOYSSR15_0903)	HG type 2.5.7 (SCN isolate PA5)	*G. soja* PI468916	[162]
	15; 212.1 kb interval between ss715621232 and ss715621239.	HG type 2.5.7 (SCN isolate PA5)	*G. soja* PI468916	[163]
*cqSCN-007*	18; (146.5 kb region between the SSR markers BARCSOYSSR_18_1669 and BARCSOYSSR_18_1675)	HG type 2.5.7 (SCN isolate PA5)	*G. soja* PI468916	[162]
	18; 103.2 kb interval between BARCSOYSSR_18_1669 and ss715631888.	HG type 2.5.7 (SCN isolate PA5)	*G. soja* PI468916	[163]
*cqSCN 10*	10 (Satt592, Satt331, and Sat_274)	LY1 nematode from a mass mating of SCN Race 2 (HG Type 1.2.5) females with Race 5 (HG Type 2.5)	PI567516C	[164]
*cqSCN11*	11	HG types 0, 2.7, and 1.3.5.6.7 (race 3, 5, and 14)	PI437654	[165]
		Races 2 (HG type 1.2.5.7), 3 (HG type 0 ) and 5 (HG type 2.5.7 )	PI90763	[156]

**Table 4:** List of host-nematode-aphid interaction studies.

Host	Nematode Species	Aphid Species	Effect	Chemistry	References
*Brassica nigra*	*Pratylenchus penetrans*	*Pieris rapae*	Negative effect on aphids	Increased phenolics and glucosinate levels	[206]
*Agrostis capillaris, Anthoxanthum odoratum*	Paratylenchidae, Pratylenchidae, and Dolichodoridae	*Rhopalosiphum padi* plus *Aphidius coleman*	Negative effect on aphid population/Reduced parasitoid mortality	Decreased foliar phenolic content and amino acid in phloem sap	[216]
*Plantago lanceolata*	*Pratylenchus Penetrans*	*Myzus persicae*	Negative effect on aphid population	-	[217]
*Brassica oleracea*	*Heterodera Schachtii*	*Brevicoryne brassicae*	Reduced body size of aphids	-	[248]
*Ammophila arenaria*	*Pratylenchus*, *Meloidogyne*, and *Heterodera spp.*	*Schizaphis rufula*	Nematodes and aphids negatively affect each other	Reduction of foliar nitrogen and amino acid	[249]
*Nicotiana tabacum*	*Meloidogyne incognita*	*Trichoplusia ni* and *Manduca sexta*	Positive effects on aboveground aphids	Change of foliar nicotine dynamics	[207]
*Brassica oleracea*	Nematode species dominant of Cephalobidae and Rhabditidae families	*Brevicoryne brassicae*	Negative effect on aphid density	-	[250]
*Arabidopsis thaliana*	*Heterodera schachtii*	*Brevicoryne brassicae*	No effect on aphid growth in presence of nematode/reduced number of nematodes in presence of aphids	Reduced glucosinolates in shoots	[215]
*Brassica oleracea*	*Heterodera schachtii*	*Brevicoryne brassicae*	Increase in aphid doubling time from 3.8 to 6.7 days	Reduced glucapin/Increased gluconapoleiferin and 4-methoxyglucobrassicin in leaves/Decreased amino acid and sugar in phloem	[210]
*Solanum tuberosum*	*Globodera pallida*	*Myzus persicae*	Positive effect on the reproduction of aphids	Increased SA in the leaves and suppression of JA	[209]
*Brassica nigra*	*Heterodera schachtii*	*Brevicoryne brassicae*	Lower preference of aphids/lower reproduction of aphids	Induced *PATHOGENESIS-RELATED 1 (PR1)* (SA pathway) Reduced *VEGETATIVE STORAGE PROTEIN2* (VSP2) and MYC2 (JA pathway)	[213]
*Brassica nigra*	*Meloidogyne hapla*	*Brevicoryne brassicae*	Higher preference of ahids/higher reproduction	No PR1 expression/High VSP2 and MYC2 expression	[213]
*Nicotiana tabacum*	*Meloidogyne incognita*, *Tylenchorhynchus* and *Pratylenchus*	*Myzus persicae*	Reduced the abundance of aphids/*Tylenchorhynchus* was decreased on aphid infested plants/no effect on *Pratylenchus*	-	[251]
*Zea mays*	*Meloidogyne incognita*	*Ostrinia nubilalis*	Reduced nematode reproduction	-	[219]
*Solanum tuberosum*	*Globodera pallida*	*Myzus persicae*	Inhibited the hatching of eggs of nematode	Decreased fructose and glucose in the root exudates	[252]
*Solanum tuberosum*	*Meloidogyne incognita*	*Myzus persicae*	No effect on the nematodes	Decreased the root SA content	[214]
*Glycine max*	*Heterodera glycines*	*Aphis glycines*	Aphids unaffected/aphid preference	-	[211,228]
*Glycine max*	*Heterodera glycines*	*Aphis glycines*	No effect of aphid on SCN reproduction	-	[229]
*Glycine max*	*Heterodera glycines* plus *Cadophora gregata*	*Aphis glycines*	SCN reproduction increased (5.24 times) in presence of SBA and *C. gregata/*aphid population decreased by 26.4% in presence of SCN and *C. gregata* in PI88788 derived cultivar	-	[220]
*Glycine max*	*Heterodera glycines*	*Aphis glycines*	SCN eggs and females increased by 33% (1.34 times) in SCN-resistant cultivar/reduced by 50% in the SCN-susceptible cultivar.	-	[212]
